# Phosphodiesterase 3B (PDE3B) regulates NLRP3 inflammasome in adipose tissue

**DOI:** 10.1038/srep28056

**Published:** 2016-06-20

**Authors:** Faiyaz Ahmad, Youn Wook Chung, Yan Tang, Steven C. Hockman, Shiwei Liu, Yusuf Khan, Kevin Huo, Eric Billings, Marcelo J. Amar, Alan T. Remaley, Vincent C. Manganiello

**Affiliations:** 1 Cardiovascular and Pulmonary Branch, National Heart, Lung, and Blood Institute, National Institutes of Health, Bethesda, MD 20892, USA; 2 The laboratory of Computational Biology, National Heart, Lung, and Blood Institute, National Institutes of Health, Bethesda, MD 20892, USA

## Abstract

Activation of inflammation in white adipose tissue (WAT), includes infiltration/expansion of WAT macrophages, contributes pathogenesis of obesity, insulin resistance, and metabolic syndrome. The inflammasome comprises an intracellular sensor (NLR), caspase-1 and the adaptor ASC. Inflammasome activation leads to maturation of caspase-1 and processing of IL1β, contributing to many metabolic disorders and directing adipocytes to a more insulin-resistant phenotype. Ablation of PDE3B in WAT prevents inflammasome activation by reducing expression of NLRP3, caspase-1, ASC, AIM2, TNFα, IL1β and proinflammatory genes. Following IP injection of lipopolysaccharide (LPS), serum levels of IL1β and TNFα were reduced in PDE3B^−/−^mice compared to WT. Activation of signaling cascades, which mediate inflammasome responses, were modulated in PDE3B^−/−^mice WAT, including smad, NFAT, NFkB, and MAP kinases. Moreover, expression of chemokine CCL2, MCP-1 and its receptor CCR2, which play an important role in macrophage chemotaxis, were reduced in WAT of PDE3B^−/−^mice. In addition, atherosclerotic plaque formation was significantly reduced in the aorta of apoE^−/−^/PDE3B^−/−^and LDL-R^−/−^/PDE3B^−/−^mice compared to apoE^−/−^and LDL-R^−/−^mice, respectively. Obesity-induced changes in serum-cholesterol were blocked in PDE3B^−/−^mice. Collectively, these data establish a role for PDE3B in modulating inflammatory response, which may contribute to a reduced inflammatory state in adipose tissue.

Insulin resistance, arthritis, asthma and obesity are associated with systemic inflammation which is characterized by increased cytokine and chemokine production and activated inflammasomes^1^,^2^. Similarly, fasting reduces inflammation in overweight adults. Adipose tissue macrophages (ATMs), and a wide variety of immune cells including T cells, B-cells and monocytes, infiltrate adipose tissue and increase the production of pro-inflammatory cytokines which play important roles in the contribution of adipose tissue to the development of obesity and insulin resistance^3^.

Release of inflammatory mediators from adipocytes may also contribute to inflammation^4^. Increased fat mass associated with obesity leads to enlargement of adipose tissue. Crosstalk among enlarged adipocytes (which are less responsive to insulin), macrophages, and activated endothelial cells perpetuate a vicious cycle of macrophage infiltration mediated by monocyte chemoattractant protein (MCP-1) and aggravate the inflammatory state^5^,^6^. The NLRP3 inflammasome, a reactive oxygen species-sensitive and oxidized mtDNA (mitochondrial DNA)-bound multi-protein complex, regulates IL-1β maturation and provides the protein scaffolds required to activate proinflammatory pathways through caspase-1 activation^2^,^6^,^7^. Mitochondrial dysfunction and generation of reactive oxygen species are implicated in cellular stress, leading to activation of NLRP3 inflammasome and insulin resistance^8^. The assembly of the NLRP3 inflammasome involves the interaction of pyrin domains of NLRP3 and ASC [apoptosis-associated, speck-like protein containing a C-terminal CARD (Caspase Activation Recruitment Domain)], and CARD-CARD interactions of ASC with procaspase-1^7^. The adipose tissue macrophages (ATMs) can be classified into M1 pro-inflammatory classically activated macrophages and M2 anti-inflammatory macrophages^3^,^9^ . In adipose tissue, the NLRP3 inflammasome promotes classical M1 macrophage activation, leading to inflammation and metabolic diseases^9^,^10^. Mice lacking key genes of the inflammasome, such as ASC, NLRP3, and caspase-1, are defective in maturation and secretion of IL1β and IL18^11^, and are protected from adipocyte hypertrophy, hyperinsulinemia, high-fat diet weight gain, and obesity-induced insulin resistance^4^,^6^,^7^. Mice with reduced expression of NLRP3 are protected from diet-induced insulin resistance, correlating with the reduced activation of T cells in adipose tissue. Loss of TNFα or IL-1β or treatment with caspase-1 inhibitor also substantially improves insulin sensitivity^4^,^12^. Consistent with these data, studies in clinical trials have shown that IL1β signaling blockade using anakinra (recombinant human IL1 receptor antagonist) leads to improvement in type-2 diabetes (T2D) and inflammation^13^. In human studies, treatment of T2D individuals with thiazolidinediones (insulin-sensitizers), reduced ATMs and inflammatory factors, and improved insulin resistance^14^. An anti-diabetic drug (sulfonylurea glyburide) has been shown to act as an inhibitor of NLRP3^15^, suggesting that NLRP3 inflammasome may be a promising therapeutic target in T2D clinical trials. Thus, WAT contributes not only to modulation of energy utilization and homeostasis, but also to metabolic dysregulation that characterizes insulin resistance and obesity-related metabolic and cardiovascular complications.

The PDE superfamily contains 11 structurally-related and functionally distinct PDE gene families (PDEs 1-11)^16^. The PDE3 family includes PDE3A and PDE3B, which are generated from two similarly organized genes, hydrolyze cAMP and cGMP, and are specifically inhibited by milrinone, cilostamide and cilostazol^16^. In general, PDE3B isoforms are relatively more highly expressed than PDE3A in tissues that regulate energy homeostasis, including adipose tissue, liver, and pancreatic β cells^16^,^17^. In adipocytes, insulin-induced activation of PDE3B mediates some acute metabolic actions of insulin, including inhibition of hormone-sensitive lipase and, thereby, hydrolysis of stored triglycerides^16^,^17^. Increased cAMP/ protein kinase A signaling is most likely responsible for reduced expression of proinflammatory genes^18^ and PKA-mediated suppression of NF-κB plays a role in controlling peripheral T lymphocytes, important in inflammation^19^. cAMP binding to NLRP3 promotes its ubiquitination and degradation^20^,^21^. However, the impact of long-term alteration of PDE3B expression on regulation of NLRP3 inflammasome and PDE3B regulation of insulin sensitivity remains to be determined.

Here we show that targeted inactivation of the murine PDE3B gene resulted in decreased activation of the NLRP3 inflammasome in epididymal white adipose tissue (WAT) as reflected in decreased protein expression of NLRP3, procaspase-1, apoptosis-associated speck-like protein (ASC), interferon-inducible protein AIM2, decreased production of IL-1β. Furthermore, ablation of PDE3B, reduced the expression of WAT proinflammatory cytokine TNFα, MAPK signaling, and atherosclerotic plaque formation. Collectively, these data establish that ablation of PDE3B leads to decreased inflammasome which may contribute to the decreased proinflammatory state and increased insulin sensitivity in adipose tissue.

## Results

### Changes in PDE3B^−/−^epididymal white adipose tissue (WAT) transcriptome

**Figure 1:**
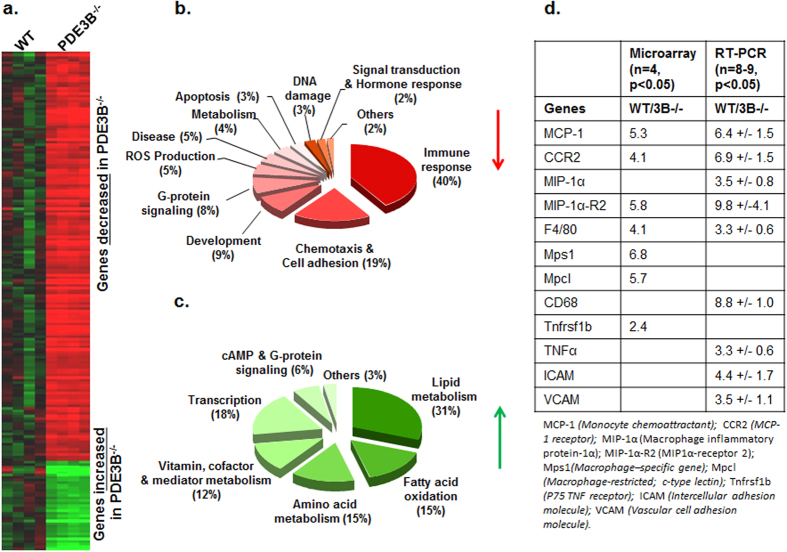
Inflammation-related gene expression was reduced in PDE3B^−/−^EWAT. (**a**) A total of 573 genes were identified as significant (*p*≤0.05 vs WT mice) level using a Welch’s t-test. Of these genes, 195 had greater than a two-fold decrease (160 genes) or increase (35 genes) in PDE3B^−/−^EWAT, compared to WT. **(b,c)** Classification of genes: decreased **(b)** or increased **(c)** in PDE3B^−/−^EWAT identified in Affymetrix microarrays (*n* = 4, 7 months old males, p < 0.05 vs WT mice), were presented as pie graphs. **(d)** Reduced proinflammatory gene expression in PDE3B^−/−^EWAT identified in Affymetrix microarrays (*n* = 4; *p < 0.05 *vs.* WT mice), 7 months old males. The ratios (WT/ KO) of mRNA transcripts for some key proinflammatory genes in EWAT were further analyzed by Real-time qPCR (arbitrary units) (*p* < 0.01, *n* = 8–12).

Upon analysis of Affymetrix MU74A Version 2 arrays, changes in expression of a total of 573 WAT genes were identified as significant (*p*≤0.05) (Table S1). Of these, 195 had greater than a two-fold increase (thirty five) or decrease (one hundred sixty) in PDE3B^−/−^WAT compared with WT WAT (Fig. 1a). Among the 35 upregulated genes in PDE3B^−/−^WAT (Fig. 1a and Table S1), expression of the UCP-1 gene, considered to be almost exclusively expressed in brown adipose tissue (BAT), is significantly increased. As seen in Fig. 1c, the largest gene subclass (31% of the 35 upregulated genes) increased in PDE3B^−/−^WAT was linked to lipid metabolism, and 15%, to fatty acid oxidation (FAO)-related genes. These results are consistent with recruitment/differentiation of brown adipocytes in PDE3B^−/−^WAT with an increase in cAMP, with coordinated upregulation of thermogenesis and energy metabolism^16^,^17^,^22^,^23^. The decreased expression of some of the proinflammatory genes in PDE3B^−/−^WAT (Table S1), and others, was verified by quantitative real-time PCR (qRT-PCR) (Fig.1d).

The majority of genes decreased in PDE3B^−/−^WAT were related to inflammation (Fig. 1b). The reduced expression of some of these genes (Fig. 1d), including CCL2 (monocyte chemoattractant protein-1, MCP-1), CCR2 (MCP-1 receptor), macrophage inflammatory protein-1α (MIP-1α) receptor2, F4/80 (EMR1), macrophage-expressed gene 1 (Mpeg1), and macrophage-specific gene (Mps1), might reflect reduced monocyte/macrophage infiltration/differentiation in PDE3B^−/−^WAT. Taken together, data in this report, as well as our previously published data^23^, are consistent with PDE3B^−/−^WAT exhibiting increased energy dissipation and reduced expression of pro-inflammatory genes in PDE3B^−/−^WAT compared to WT.

### Reduced expression of components of NLRP3 inflammasome in PDE3B^−/−^mice adipose tissue

**Figure 2:**
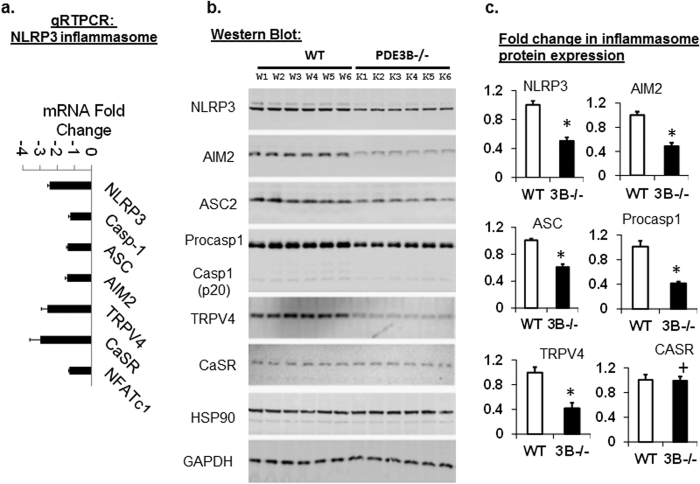
Reduced expression of NLRP3 inflammasome in PDE3B^−/−^mice adipose tissue determined by qRT-PCR and Western blot. **(a)** Real time quantitative PCR analysis of NLRP3 inflammasome (NLRP3, Caspase-1, ASC, AIM2, TRPV4, CaSR, NFATc1) mRNA gene expressions in WAT of 3–4 month old WT and PDE3B^−/−^SvJ 129 mice fed on standard chow (SD) diet (n = 18 mice per group). **(b,c)** NLRP3 inflammasome protein expressions were detected by Western blotting **(b)** in adipose tissue lysates from WT and PDE3B^−/−^mice. Quantification of relative protein expressions **(c)** were determined by Western blot data from at least two independent experiments and plotted in the graph as fold change. There was a significant decrease in the expression of NLRP3 inflammasome proteins in PDE3B^−/−^mice adipose tissue compared to WT controls. GAPDH protein levels are shown as loading controls. (*p < 0.01 vs WT, +p = ns vs WT, n = 2 independent experiments, each experiment with samples from 6 WT and 6 PDE3B^−/−^adipose-tissue lysates). W, Wild-type; K, PDE3B-Knockout.

**Figure 3:**
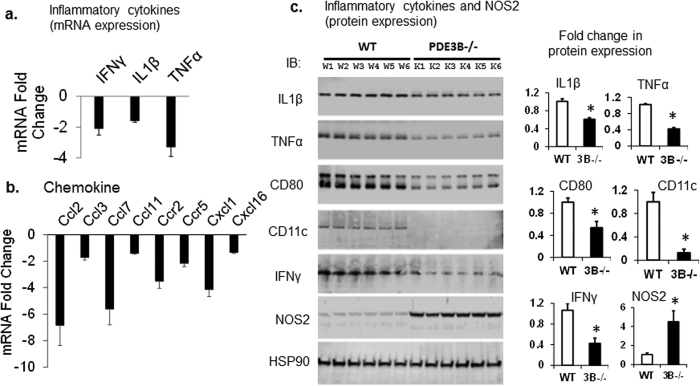
Adipose tissue inflammation and gene expression in PDE3B^−/−^mice. WAT was collected from SvJ129 WT and PDE3B^−/−^mice. **(a)** mRNA expressions of inflammatory cytokines (IL1β, TNFα, IFNγ) were analyzed using qRT-PCR. (N = 5 WT and 5 PDE3B-KO mice) and presented as fold-up/down-regulation of expression of mRNA levels. **(b)** Downregulation of major inflammatory chemokines. Experiment conducted using Mouse Inflammasome Profiler PCR Array (Qiagen) and depicted here as fold-up/down-regulation of mRNA levels. Results are presented as relative mRNA fold change (fold change+/−SE) compared to WT mice (N = 4 WT and 4 PDE3B^−/−^mice). **(c)** Protein expressions of inflammatory cytokines (IL1β, TNFα, IFNγ), CD80, CD11c and NOS2 were analyzed using Western blots. HSP90 protein levels were used as loading control. Results are presented as relative protein fold change compared to WT mice. All bar graphs represent fold change+/−SE (n = 2 independent experiments, each experiment with samples from 6 WT and 6 PDE3B^−/−^ mice adipose tissue lysates).

Since the NLRP3 inflammasome is an important regulator of inflammation and the production of inflammatory cytokines (IL1β and IL18), we analyzed the expressions of components of NLRP3 inflammasome in WT and PDE3B^−/−^mice adipose tissue. As determined by qRT-PCR (Fig. 2a), the relative gene expression of NLRP3 is reduced by more than 2-fold in PDE3B^−/−^WAT. The mRNA expression levels of several inflammasome components-[caspase-1, ASC (PYCARD), AIM2, trpv4 (transient receptor potential vanilloid 4), CaSR and NFATc1] are reduced in PDE3B^−/−^mice adipose tissue compared to WT littermates (Fig. 2a). Gene expression of CasR (calcium-sensing receptor, which regulates NLRP3 inflammasome through Ca2+ and cAMP)^21^ is decreased, while CaSR protein expression is unchanged (Fig. 2b,c). Western blot analysis revealed that protein levels of procaspase-1 (45 kD) and caspase-1 (20 kD) are significantly lower in PDE3B^−/−^than WT adipose tissue (Fig. 2b,c). Similarly, the protein expressions of NLRP3 and its component inflammasome (AIM2, ASC) and proinflammatory mediator COX2 (cyclooxygenase 2), which catalyzes the release of IL1β (Fig. 3c), are reduced in PDE3B^−/−^mice adipose tissue. Analysis of serum IL1β concentration in WT and PDE3B^−/−^mice treated with LPS (lipopolysaccharide) for 0 h, 6 h, and 24 h indicates significantly reduced levels of the cytokine in PDE3B^−/−^mice compared to WT littermates at 6 h and 24 h time intervals (Table 1). These data suggest that PDE3B^−/−^mice have reduced levels of NLRP3 inflammasome.

### PDE3B deletion promotes an anti-inflammatory phenotype in adipose tissue

To observe the effects of PDE3B on macrophage polarization in adipose tissue, the gene expressions of proinflammatory cytokines (IFNγ, IL1β, TNFα) that are characteristic of M1 or “classically activated” macrophages and chemokines were evaluated. mRNA expressions of proinflammatory M1 marker cytokines (Fig. 3a), and chemokines (Fig. 3b) are decreased in PDE3B^−/−^mouse adipose tissue. In mice, diet-induced obesity (DIO) is associated with accumulation of CD11c+ cells in adipose tissue^10^. CD11c is found mostly on dendritic cells, functioning primarily in phagocytosis. Western blotting data suggest reduced expression of CD11c and CD80 (Fig. 3c). Expressions of inflammatory cytokines (IL1β, TNFα, IFNγ) are also decreased in PDE3B^−/−^adipose tissue (Fig. 3c). Quantification of protein expression levels of inflammatory cytokines suggest a significant decrease in PDE3B^−/−^adipose tissue compared to WT controls. In addition, increased expression of NOS2 in PDE3B^−/−^adipose tissue may contribute to increased NO (Fig. 3c), which inhibits caspase-1, preventing release of IL1β and IL18^24^, and inhibit NLRP3 inflammasome activation^25^. These data demonstrate that a significant amount of ATMs was decreased in PDE3B^−/−^mouse adipose tissue. Treatment of Type 2 diabetic individuals with insulin sensitizers (Thiazolidinediones) showed a corrrelation between reduced ATMs and inflammation and improved insulin resistance^14^.

### Modulation of NFkB, NFAT and smad signaling in PDE3B^−/−^mice adipose tissue

**Figure 4:**
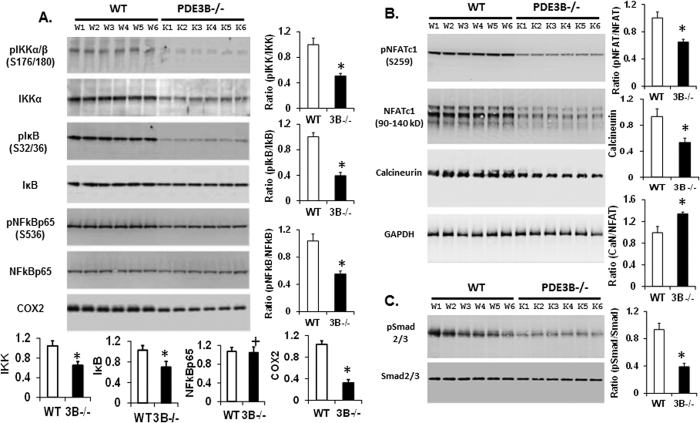
Modulation of NFκB, NFAT and SMAD signaling in PDE3B^−/−^mouse adipose tissue. (**A**) Reduced NFkB signaling: IKK, IkB, NFkB proteins and their phosphorylated forms were detected as shown by Western blot in adipose tissue lysates from WT and PDE3B^−/−^mice. Relative expression of IKK, IkB, NFkBp65, COX2 and ratios of protein phosphorylation/total protein (pIKK/IKK, pIkB/IkB, pNFkB/NFkB) was determined by quantification of Western blot data from at least two independent experiments and plotted in the graph. Ratios of pIKK/IKK, pIkB/IkB and pNFkB/NFKB were decreased in PDE3B^−/−^mice adipose tissue. (**B)** Reduced NFAT signaling: NFAT proteins are activated by increase in intracellular calcium leading to activation of calcineurin (calmodulin-dependent phosphatase), dephosphorylating NFAT. Relative expression of calcineurin and ratios of pNFAT/NFAT, NFAT/CaN from quantification of Western blot data are presented. (**C**) Smad2/3 phosphorylation and protein expressions were analyzed by immunoblotting. Ratio of pSmad2/3/ Smad2/3 was analyzed from quantification of at least two independent experiments and plotted in the graph. There was a significant decrease in the relative phosphorylations of pIkB, pNFkB, and pSmad2/3 in PDE3B^−/−^mice adipose tissue in comparison to the WT controls (**A–C**). NFkB and GAPDH protein levels are shown as loading controls. All bar graphs represent mean+/−SE, n = 2 independent experiments, each with samples from 6 WT and 6 PDE3B^−/−^ mice adipose tissue lysates) (*p < 0.01 vs WT).

In unstimulated state, NFkB is sequestered in the cytoplasm by IkB inhibitory proteins^26^. NF-kB/Rel complexes are further activated by phosphorylation, acetylation, and glycosylation. Free and active NFκB translocates to the nucleus and activates transcription of proinflammatory cytokines and enzymes involved in the generation of proinflammatory mediators (COX2)^27^,^28^. IKKα/IKKβ serve as catalytic subunits and IKKγ as the regulatory subunit^29^. Activation of IKKα/β and IkB depends upon the phosphorylation of Ser176/177 and Ser32/36, respectively^29^. In PDE3B^−/−^mice adipose tissue, the ratios of pIKK/IKK, pIkB/IkB and pNFkB/NFkB were significantly reduced (Fig. 4A). Expression of COX2, which catalyzes the synthesis of prostaglandin-E2 (mediators of inflammation) and increases IL-1β secretion, is also reduced in PDE3B^−/−^mice adipose tissue (Fig. 4A). In response to LPS challenge, inhibition of COX-2 in mice with COX2 inhibitor celecoxib reduced serum IL-1β and caspase-1 activity and regulate the NLRP3 inflammasome^30^. Similar to NFkB, Nuclear Factor of Activated T cells (NFAT) family of transcription factors has a Rel Homology Domain (RHD) and regulates immune responses. NFAT proteins are phosphorylated and located in cytoplasm in resting cells. Increased intracellular calcium concentration activates the calcium/calmodulin-dependent serine phosphatase calcineurin (CaN), resulting in dephosphorylation of NFAT and its translocation to the nucleus^31^. NFAT signaling terminates upon decrease in calcium concentration and phosphorylation of NFAT^32^. Ratios of pNFAT/NFAT and expression of calcineurin are decreased in PDE3B^−/−^adipose tissue (Fig. 4B). Western blot quantification data suggest that, although calcineurin and NFAT protein expressions decreased in PDE3B^−/−^adipose tissue, the ratio of CaN/NFAT increased, suggesting relatively decreased expression of NFAT than calcineurin, contributing to increased dephosphorylation of NFAT in PDE3B^−/−^mice adipose tissue.

Activation of transforming growth factor-β (TGFβ) receptors leads to phosphorylation of Smad2/3 and their subsequent translocation to the nucleus and transcription of target genes^33^. TGFβ levels positively correlate with obesity^34^,^35^ and Smad3^−/−^mice are protected from high fat diet (HFD)-induced obesity and diabetes^36^. We examined the phosphorylation of Smad2/3 (pSmad2/3) to evaluate the activation of TGF-β signaling in WAT. The total lysates from WT and PDE3B^−/−^WAT were subjected to Western immunoblotting using antibody against pSmad2/3. As shown in Fig. 4C, ratio of pSmad/Smad is reduced in PDE3B^−/−^WAT. Reduced ratio of pSmad/Smad in PDE3B^−/−^WAT (Fig. 4C) suggest that PDE3B regulation of TGF-β/smad signaling may play a role in the effects of smad signaling in the pathogenesis of obesity, inflammation and development of insulin resistance.

### Reduced MAP kinase pathway signaling in PDE3B^−/−^WAT

**Figure 5:**
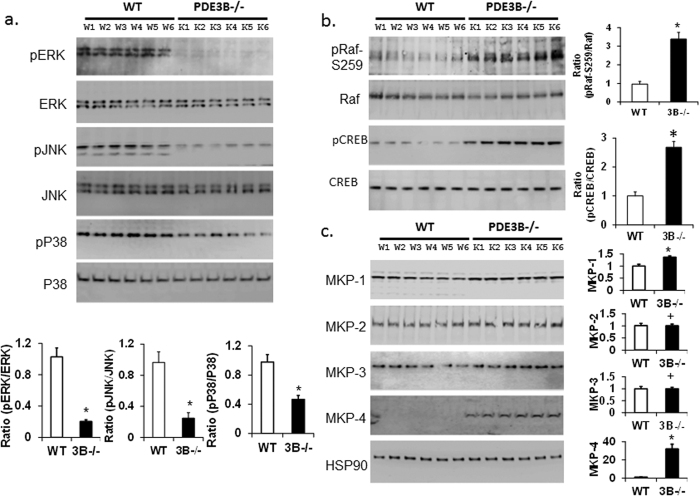
Down-regulation of PDE3B inhibits the MAP kinase pathway at the levels of Raf-1 and MAP kinase Phosphatases (MKP). (**a–c**) Phospho-ERK1/2 (pERK), total ERK; phospho JNK, total JNK; phospho-p38, total p38 (**a**); pRaf1, total Raf1; pCREB, total CREB (**b**); and MKP1-4 (**c**); Protein expression and their phosphorylation levels were detected in adipose tissue lysates from WT and PDE3B^−/−^mice. Ratios of phospho/total proteins (**a,b**), and the relative expression of MKP1-4 (**c**), were determined using quantification of Western blot data from at least two independent experiments (each with adipose tissue samples from 6 WT and 6 PDE3B^−/−^ mice lysates) and plotted in the graph. Data are mean+/− SE (*P  < 0.01, +P = NS, compared to WT control). There was a significant decrease in the phosphorylation of MAPKs (ERK, JNK, and P38) in PDE3B^−/−^mice adipose tissue compared to WT controls. HSP90 protein levels are shown as loading control.

Mitogen-activated protein kinases (MAPKs) are a family of conserved serine/threonine protein kinases (ERK, p38 and JNK) and regulate multiple cellular processes including cellular stress and inflammation. To test the possibility that inhibition of NLRP3-inflammasome might be related to the alterations in the MAP kinase signaling, Western immunoblot analysis was performed on WAT lysates (Fig. 5a). Studies in PDE3B^−/−^WAT suggest that phosphorylations of MAPKs (e.g. ERK, JNK and p38) was significantly reduced (Fig. 5a). Western blot densitometric analysis show reduced phosphorylation ratios of pERK/ERK, pJNK/JNK and pP38/P38 of MAPKs in PDE3B^−/−^WAT compared to WT. Reduced phosphorylation of MAPKs was not due to reductions in protein expression. In MAPK pathway, Raf-1 phosphorylates and activates MEK, which then phosphorylates and activates ERK. The cAMP/PKA signaling increases phosphorylation of Raf-1 at Ser259, resulting in inhibition of Raf-1 kinase activity^37^. As shown in Fig. 5b, Raf1-S259 inhibitory-site serine-phosphorylation was significantly elevated (more than 3 fold) in PDE3B^−/−^WAT compared to wild-type WAT, which is consistent with increased PKA activity and CREB phosphorylation (Fig. 5b). As shown in Fig. 5c, densitometric analysis of immunoblots suggested a significant increase in MKP-1 protein expression in PDE3B^−/−^WAT. Earlier studies also indicated that increased cAMP/PKA signaling induces MKP-1 expression^38^. Immunoblotting studies suggest a low level expression of MKP-4 in WT adipose tissue. In PDE3B^−/−^mice, expression of MKP-4 was increased more than 20 fold (Fig. 5c). However, expressions of MKP-2 and MKP-3 did not change in PDE3B^−/−^WAT. Thus, in PDE3B^−/−^WAT, increased cAMP/PKA signaling appears to induce the expressions of MKP-1 and MKP-4, resulting in enhanced dephosphorylation and inactivation of MAPKs and reduced MAPK signaling.

### Increased resistance of PDE3B^−/−^mice to lipopolysaccharide-induced shock

The decreased immune cell infiltrations in WAT of HFD-fed PDE3B^−/−^mice led us to investigate serum cytokine levels in these mice. We examined the role of PDE3B in a low-dose LPS-induced shock in WT and PDE3B^−/−^mice. Endotoxin shock in mice is mediated by a complex array of pro- and anti-inflammatory cytokines and chemokines. Levels of these cytokines/ chemokines increased in the serum within hours after LPS injection. Wild-type and PDE3B^−/−^mice (6~8-month-old) were treated (ip injection) with LPS (2.5 mg/kg) for 6 h and 24 h (4 mice per each group) and serum levels of cytokines and chemokines were determined (using Bio-Plex Mouse Cytokine 23-plex immunoassay kit). We did not find any differences in the basal levels of cytokines (Table 1). However, our results suggest a role of reduced PDE3B expression in the regulation of LPS-induced cytokine at 6 h and 24 h after LPS injection (Table 1). Serum levels of inflammatory cytokines (IL1β, TNF-α, MCP-1, MIP-1β and IL12) were significantly inhibited in PDE3B^−/−^mice after 6 h IP injection compared to WT mice (Table 1). LPS-induced changes in the cytokine levels were mostly normalized after 24 h of injection. After 24 h, serum cytokines (MCP-1 and MIP-1) levels were still significantly lower in PDE3B^−/−^than in their wild-type littermates. However, levels of some cytokines/chemokines (IL-6, IL-10, GM-CSF) were similar between wild-type and PDE3B^−/−^mice in response to LPS, suggesting selective regulation of cytokines. These studies suggest that PDE3B participates in LPS-induced shock by regulating secretion of selected proinflammatory cytokines from macrophages and point to a novel role of PDE3B in control of the proinflammatory cytokine network. Even though anti-inflammatory cytokines such as IL-10 did not change between the wild-type and PDE3B^−/−^mouse serum, the profound decrease in the selected pro-inflammatory cytokines (especially IL1β, TNFα, MCP-1, MIP-1β, and IL12) may be sufficient to change the balance towards the anti-inflammatory phenotype observed in the PDE3B^−/−^mice.

### Reduced expression of NLRP3 inflammasome is associated with reduce-macrophage infiltration and improved insulin sensitivity in PDE3B^−/−^WAT during high-fat feeding

**Figure 6:**
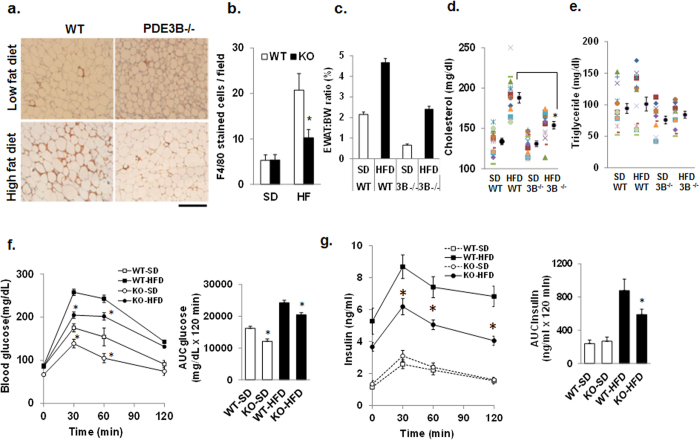
Reduced macrophage infiltration, WAT/BW ratio and adiposity in PDE3B^−/−^WAT during high fat feeding. Age-matched (8 week old) WT and PDE3B^−/−^mice were housed two per cage, with food and water ad libitum. Mice were fed high fat diets (HFD) (60 kcal%) (D12492, Research diets, NJ) and low fat standard diets (SD) (10 kcal%) (D12450B, Research diets, NJ), for 10 weeks. Protein/carbohydrate/fat contents as a percentage of caloric content were 20/70/10 kcal (%) for SD diets and 20/20/60 kcal (%) for HFD diets. **(a)** Immunohistochemical staining of macrophage-specific marker F4/80 (Emr1) in WAT from mice fed SD (10 kcal%, *n* = 5), and HFD (60 kcal%, *n* = 5), respectively (stained brown, bar = 500 μM). **(b)** F4/80-stained cells were counted in 40 fields and the average number per field calculated, mean ± SE, (n = 5, *p < 0.05). **(c)** Percent of WAT weight to body weight (BW) (5–6 week old age-matched males): WT = 2.1+/−0.11% (n = 14) and WT+ HFD= 4.7+/−0.18 (n = 14); PDE3B^−/−^ = 0.66+/−0.06 (n = 14), PDE3B^−/−^+HFD = 2.4+/−0.13 (n = 14). **(d,e)** Serum cholesterol **(d)** and triglyceride **(e)** levels were assayed in WT and PDE3B^−/−^male mice fed a SD or HFD diets. **(f,g)** Intraperitoneal GTT (2 g/kg) following fasting for 6 h (F-G), blood glucose **(f)** and insulin **(g)** levels were assayed and area under the curve for glucose (AUCglucose) and insulin (AUC insulin) were calculated using the trapezoidal rule. Data are mean+/−SE. P*  < 0.01 compared with their respective controls.

Feeding of high-fat diet (HFD) activates NLRP3 inflammasome, inducing increased production of caspase-1, IL-1β and IL18, contributing to insulin resistance and type 2 diabetes (T2D)^12^,^39^. In HFD-fed mice, expression levels of IKK£ and TBK1 are increased in adipose tissue as a consequence of inflammation^40^,^41^. Reduced expression of IKK£ gene partially protected mice from harmful effects of HFD feeding, including insulin resistance, weight gain, and inflammation^40^; knockout of IKKβ preserves insulin sensitivity through reduced expression of IL1β^42^. Earlier reports suggest that inhibition of PDE3 is therapeutically useful in mediating anti-inflammatory effects in VSMC through inhibition of NFkB^43^. Affymetrix microarray analysis (Fig. 1) identified a systematic difference in the expression profile of proinflammatory genes in PDE3B^−/−^WAT. mRNA for F4/80 (Emr1), a macrophage marker protein^5^, was decreased by 4.1 fold in PDE3B^−/−^WAT. Macrophage infiltration in WAT was analyzed via F4/80 immunohistochemical staining in mice fed with or without a high-fat diet. As shown in Fig. 6a,b, accumulation of macrophages in WAT, which was associated with high-fat feeding, was significantly reduced in PDE3B^−/−^WAT compared to WT, consistent with reduced proinflammatory molecules in PDE3B^−/−^WAT. Reduced inflammation in PDE3B^−/−^WAT may be related to reduced fat cell mass and adipocyte size^23^, which is associated with reduced infiltration of macrophages in adipose tissue, contributing to reduced inflammation. In PDE3B^−/−^mice (male), fed a 60 kcal% HFD for 10 weeks, the-percentage of WAT weight relative to body weight was significantly reduced as compared to WT littermates (Fig. 6c). Similar to these results, we recently reported that WAT mass and adipocyte size were decreased in age-matched male PDE3B^−/−^mice compared to WT, although body weight was significantly increased in PDE3B^−/−^mice^23^. Circulating serum cholesterol levels were not significantly different in PDE3B^−/−^and WT mice. Serum cholesterol levels of HFD fed PDE3B^−/−^mice were significantly lower than in WT mice (Fig. 6d). These studies suggest that PDE3B^−/−^mice are resistant to changes in their serum cholesterol levels after feeding a high-fat diet. However, serum triglycerides concentrations were not significantly different in SD- and HFD-fed PDE3B^−/−^mice and WT mice (Fig. 6e). To further understand the role of PDE3B and its effects on NLRP3 inflammasome in regulation of blood glucose, we performed intraperitoneal glucose tolerance tests (IPGTT) in WT and PDE3B^−/−^mice that had been fed a HFD or standard diet, starting 2 months of age for 10 weeks. The IPGTT revealed that the reduced expression of PDE3B and NLRP3 inflammasome led to substantial protection against HFD-induced insulin resistance (Fig. 6f,g). In SD- and HFD-fed mice, ablation of PDE3B contributes to increased insulin sensitivity (Fig. 6f,g).

**Figure 7:**
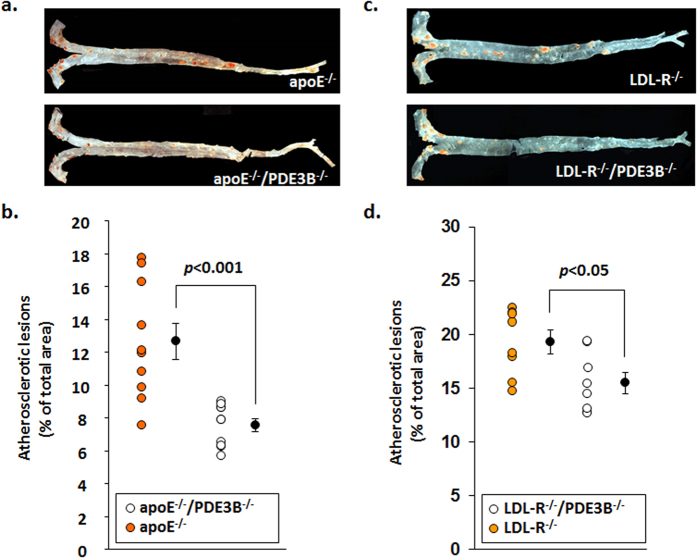
Crossbreeding of PDE3B^−/−^ with apoE^−/−^ and LDL-R^−/−^ mice (C57BL/6J) lead to a dramatic reduction of fatty streak lesions within the aorta. Plaque development in aorta. (**a,c**) The aorta was dissected from the origin of the heart to the ileal bifurcation and was then further fixed overnight with fixative solution, stained with Sudan IV solution for 25 min, destained for 25 min in 80% ethanol, and washed with clean molecular grade water. After removal of any remaining adventitial fat, the aortas were cut longitudinally, placed on a glass slide embedded with glycerin, covered by a microscope cover glass, and sealed. Slides were scanned on a Leica inverted microscope and aorta images were captured using a Metamorph software program. The surface area of the aortas and their plaques surface area from the ileal bifurcation to the origin (not including branching vessels) of each animal was quantified in a blind fashion and in triplicate, using the Image-Pro Plus version 4.1 software (Media Cybernetics, Inc., MD). (**b,d**) Data are reported as the percentage of the aortic surface covered by lesions (total surface area of the atherosclerotic lesions divided by the total surface area of the aorta), mean ± SE (apoE^−/−^/PDE3B^−/−^, *n* = 10; LDL-R^−/−^/PDE3B^−/−^, *n* = 8).

Our studies confirm that ablation of PDE3B was associated with decreased macrophage markers in WAT. Furthermore, after LPS injection, plasma levels of IL1β, TNF-α, IL-12 and CCL2/MCP-1 were lower in PDE3B^−/−^mice than WT mice (Table 1). Moreover, chemokines CCL2, MCP-1and its receptor CCR2, which play important roles in macrophage chemotaxis, were less highly expressed in WAT of PDE3B^−/−^mice than WT mice. We were interested to know if reduced expression of PDE3B will help in moderation of atherosclerosis in apoE^−/−^and LDL-R^−/−^mice. Apolipoprotein E knockout (apoE^−/−^) and low-density lipoprotein receptor (LDL-R^−/−^) mice develop hypercholesterolemia and atherosclerosis spontaneously or under a high cholesterol diet, respectively. Inflammatory infiltrates in the atherosclerotic plaques contain cholesterol-laden macrophages (“foam cells”) and T lymphocytes. To examine the possible effects of PDE3B on macrophage infiltration and atherosclerotic plaque formation, apoE^−/−^/PDE3B^−/−^, as well as LDL-R^−/−^/PDE3B^−/−^mice were generated. Compared to apoE^−/−^and LDL-R^−/−^mice, in the aorta of apoE^−/−^/PDE3B^−/−^and LDL-R^−/−^/PDE3B^−/−^mice fed standard diet (fat content 10 kcal%) and (HFD, fat content 60 kcal%) for 10 weeks, plaque formation was significantly reduced (Fig. 7), in PDE3B^−/−^crossbred mice, suggesting that reduced expression of PDE3B play a role in modulating the inflammatory response and PDE3B signaling might be a possible therapeutic target to moderate atherosclerosis.

## Discusssion

PDEs regulate concentrations of intracellular cAMP and cGMP and play critical roles in regulating physiological mechanisms^16^. The PDE3 family contains two subfamilies, PDE3A and PDE3B, which are encoded by distinct but related genes and exhibit different-, but overlapping-patterns of expression and functions in different tissues and cells. To study the effects of PDE3B on regulation of NLRP3 inflammasome in adipose tissue, PDE3B gene was inactivated in SvJ129 mice. Infiltration of ATMs (adipose tissue macrophages) in adipose tissue is associated with increased obesity, chronic inflammation, and development of insulin resistance^3^. PDE3B plays a key role in the regulation of lipolysis, energy homeostasis and insulin secretion through activation of cAMP/PKA-signaling. In HFD-fed mice, PDE3B overexpression cause diabetes-like symptoms, suggesting a role for cAMP/PKA signaling in regulation of HFD-induced insulin resistance^44^. PDE3B KO mice also show beneficial long-term factors, which included smaller adipocytes and reduced amounts of white adipose tissue and increased lean mass than Wild-type control mice^16^,^22^,^23^. These changes may contribute to the reduced infiltration of macrophage content and inflammation. In PDE3B^−/−^mice adipose tissue, increased expression of NOS2 (Fig. 3), which inhibits NLRP3-mediated processing of IL1β through inhibition of caspase-1^25^,^45^ (Fig. S1), reduced expressions of TRPV4 (regulator of inflammation, oxidative metabolism and cellular respiration)^46^, and NFATc1 (Figs 2a and 4B), contributing to deactivation of NLRP3 inflammasome^20^,^21^,^47^ (Fig. S1). WAT contains adipocytes and stromal-vascular cells including macrophages, leukocytes, fibroblasts and pre-adipocytes. Due to the presence of adipocytes and immune cells in WAT, effects of PDE3B-knockdown on reduced NLRP3-inflammasome may not be a direct adipocyte-mediated phenomenon.

In PDE3B^−/−^mice, increased phosphorylation of CREB and cAMP/PKA-signaling inhibit Raf1 kinase activity by increasing the phosphorylation of Raf-1 at Ser-259^37^, and consequently down-regulate MAPK phosphorylation/activation (Fig. 5). Proinflammatory cytokines (i.e. TNFα, IL1, IL6) activate all three classes of MAPKs^48^. Inhibition of IL1 reduced the activation of p38-MAPK and blockade of TNF reduced the activation of ERK and p38-MAPK^49^. As assessed by RT-PCR and Western blots, inflammatory cytokines and chemokines mRNA and protein expressions were decreased in PDE3B^−/−^adipose tissue (Fig. 3). In adipose tissue from PDE3B^−/−^mice, COX2 expression, NFkB signaling, and smad2/3 phosphorylations are significantly reduced, contributing to reduced inflammation (Fig. 4). Similarly, following LPS challenge, serum inflammatory cytokine levels were also reduced in PDE3B^−/−^mice compared to their WT littermates (Table 1). In addition, MAPKs phosphorylation/activation was reduced possibly due to PKA-mediated induction of MKP-1^38^ and MKP-4 in adipose tissue from PDE3B^−/−^mice, which catalyze dephosphorylation of MAPKs (Fig. 5). In PDE3A-KO mice VSMCs, MAPK phosphorylation/ activation was reduced due to increased expression of MKP-1 and dephosphorylation of MAPK^50^. This impairment in MAPK activation was specific to PDE3A deficiency as PDE3B^−/−^mice VSMCs exhibited no elevations in PKA activity^50^. The specificity in the impairment of MAPK activations in different tissues in PDE3A and PDE3B knockout mice may be provided due to differences in the expressions of PDE3A and PDE3B in these tissues, with relatively higher expression of PDE3A in the cardiovascular system and of PDE3B in cells important in the regulation of glucose and lipid metabolism^16^,^50^.

Diet-induced obesity and inflammatory mediators such as LPS and IFNγ induce M1 macrophages and enhance inflammatory cytokine production (TNFα) and activate iNOS (NOS2) generating reactive oxygen species^10^. The density of ATMs in adipose tissue correlates with the expression of inflammatory markers (e.g. TNFα, IL1β) that contribute to increased inflammation and potentiation of insulin resistance^7^,^39^. In the current study, on both chow- and HFD-fed mice, WAT macrophage infiltration (Fig. 6) and expression of inflammatory cytokines (Fig. 3) is decreased. These data suggest that PDE3B deficiency can suppress many important WAT inflammatory responses induced by HFD treatment. In HFD-fed PDE3B^−/−^mice, serum cholesterol levels were significantly lower than in their WT littermates (Fig. 6). In ApoE^−/−^and LDL-R^−/−^mice (Fig. 7), atherosclerotic plaque formation was significantly increased due to increased serum inflammatory cytokines and incorporation of M1-macrophages into atherosclerotic plaques. In apoE-/-/PDE3B^−/−^as well as LDL-R-/-/PDE3B^−/−^mice, increased cAMP/PKA signaling with decreased expression of PDE3B resulted in reduced macrophage infiltration and atherosclerotic plaque formation, due to reduced inflammatory serum cytokines and reduced inflammation. These studies suggest that reduced expression of PDE3B play an important role in reduced atherosclerotic plaque formation and regulation of serum cholesterol in HFD-fed mice. The intraperitoneal (IP) glucose tolerance test (GTT) in PDE3B^−/−^mice (Fig. 6), revealed that reduced NLRP3 inflammasome together with reduced inflammatory cytokines, chemokines, and cholesterol may be responsible for improved insulin sensitivity in PDE3B^−/−^mice compared to their WT littermates.

Studies in VSMC have shown that PDE3A is a novel mediator of inflammation^43^. To our knowledge, our current findings may define, for the first time, roles of PDE3B in the regulation of the NLRP3 inflammasome in adipose tissue through increased cAMP/PKA signaling^20^,^21^ (Fig. S1). NLRP3 inflammasome specifically regulates activation of caspase-1 (IL1- converting enzyme), a protease responsible for the processing of the key pro-inflammatory cytokine IL-1*β* from an inactive precursor to an active, secreted cytokine, which amplify inflammatory responses^51^. Our current data show that decreased expression of NLRP3 inflammasome in PDE3B^−/−^mice adipose tissue reduces the expression of M1-like macrophages and suggests that the NLRP3 inflammasome participates in the induction of inflammation by inducing macrophage and T cell activation.

In summary, PDE3B-deficient mice have reduced NLRP3 inflammasome activation in adipose tissue, which may be a result of increased cAMP/PKA-signaling, with an associated increase in insulin sensitivity. Importantly, HFD-fed PDE3B^−/−^mice show suppressed macrophage infiltration. Our data establish that NLRP3 inflammasome-mediated increase of IL1β, TNFα and IL12-p40 in response to LPS-associated danger signals participate in the development of proinflammatory state, leading to insulin resistance. Thus, cAMP/PKA-signaling regulated by PDE3B in adipose tissue and potential targeting of caspase-1 activation through NLRP3 inflammasome, may be a new therapeutic target for the development of anti-obesity and anti-inflammatory drugs.

## Materials and Methods

### Antibodies

Antibodies for immunoblotting were obtained as follows from specified commercial sources with their catalog numbers in parentheses: from Cell Signaling Technology (Beverly, MA): IkB (9242s), pIKKα/β-S176/180 (2694P), pSmad2/3 (8828), NFkB-p65 (8242), P38-MAPK (9212), pNFkB-p65 (3033), TNFα (3707); from Santa Cruz Biotechnology (Santa Cruz, CA): ASC1 (SC-365202), Caspase-1 (SC-56036), HSP90 (SC-13119), pIkB-S32/36 (SC-101713), NFATc1 (SC-13033), pNFATc1-S259 (SC-32979), MKP1 (SC-1199), MKP3 (SC-377070), MKP4 (SC20463), pRaf1-S259 (SC-101791); from BD Biosciences, Inc (San Jose, CA): ERK-MAPK (610031), pERK-pT202/pY204 (612359), pP38-pT180/pY182 (612281), MKP2 (610850), Smad2/3 (610843), Raf1 (610152), Cyclooxygenase-2 (COX2) (610204), JNK (610628), pJNK-pT183/pY185 (612541); from Protein Tech Group (Chicago, IL): TRPV-4 (20987-1-AP); from Abcam (Cambridge, MA): CD80 (ab-64116), CaSR (ab-18200); from Novus Biologicals (Littleton, CO): IL1β (NB600-633); from Thermo Scientific (Waltham, MA): IFNγ (MM701); Acris Antibodies Inc (San Diego, CA): NLRP3 (AP22017PU-N); EMD Millipore (Billerica, MA): IKKα (OP133); GeneTex (Irvine, CA): GAPDH (GTX627408); AbD Serotec (Bio-Rad): F4/80 (MCA497R); R&D Systems, Inc (Minneapolis, MN): CD11c (MAB6950). Rabbit polyclonal antibodies against mouse PDE3B (GenBank^®^ accession number AAN52086) were generated against peptides corresponding to the C-terminal (CT) domain (amino acids 1076–1095; NASLPQADEIQVIEEADEEE), and the N-terminal (NT) domain (amino acids 2–16; RKDERERDAPAMRSP)^52^. Affinity-purified anti-PDE3B-NT and anti-PDE3B-CT antibodies were used for Western blotting.

### cAMP PDE assay

PDE activity was measured as described previously^52^. Samples (usually 5~15 μg protein in 100 ul) were incubated for 10~15 minutes in a total volume of 0.3 ml assay buffer containing 50 mM HEPES, pH 7.4, 0.1 mM EGTA, 8.3 mM MgCl2, and 0.1 μmol/L [^3^H]-cAMP. PDE3 activity [that portion of total PDE activity inhibited by 1.0 *μ*M cilostamide (calbiochem, cat#231085), a selective PDE3 inhibitor (IC_50_ ~17–80 nM)]. Inhibitor vehicle, DMSO, added in equal quantities to samples without inhibitors, did not-alter PDE activity. Protein concentration was determined by BCA protein assay.

### Glucose tolerance tests GTT and blood glucose measurements

Prior to a glucose challenge, animals were fasted for 6 h (6 am to 12 pm, while individually housed in a clean cage with cotton bedding. Glucose obtained from Amresco Inc (Solon, Ohio) and formulated in a sterile-saline solution (20% glucose). The glucose dose was adjusted by fasted body weight and administered by a single intraperitoneal (ip) injection of 2 g/kg. Blood glucose was measured using TRUEresult^®^ meters (Nipro diagnostics) from blood droplets obtained from a small nick at the tip of the tail, at time points 0, 30, 60 and 120 min with reference to the time of glucose injection. The area of the blood glucose response profile curve corresponding to each animal was calculated by the trapezoid method^53^, using as reference each individual baseline blood-glucose measurement prior to glucose administration (t = 0) or the lowest point of the curve. The sum of the trapezoidal areas between the 0, 30, 60 and 120 minute time points corresponding to each animal were summed to obtain the area under the curve (AUC). The relative area values are expressed as a percentage relative to the average AUC of the vehicle, which is defined as 100%. Values are reported as mean ± SE Statistics were performed using a two-tailed Student’s t-test.

### PDE3B KO mice

PDE3B KO mice were progeny of 7-10 backcrosses of heterozygous (HE) F1 mice with JAX 129/SvJ (pTyr^c-ch^/pTyr^c^) substrain^23^. With primers from PDE3B exons 2/3 and 8/9, mRNA amplification in PDE3B^−/−^WAT was ~5% that of wild type (WT). With primers 3′ to exon 9, i.e. exons 9/10 and 15/16, mRNA amplification was ~15% that of WT^23^. Crossbreeding of PDE3B^−/−^in C57BL/6 background with ApoE^−/−^and LDL-R^−/−^mice were described in the supplemental section. Mice were maintained, and studies performed, in accord with protocols approved by the NHLBI Animal Care and Use Committee.

### Measurement of cytokines

Wild type (W) and PDE3B^−/−^(K) mice (6~8-month-old) were treated (ip injection) with LPS (2.5 mg/kg) for 6 h and 24 h (4 mice per each group). Paired serum samples from untreated mice (*C*) and mice treated with LPS were stored at -80 °C and were analyzed using Bio-Plex Mouse Cytokine 23-plex immunoassay kit and the Luminex100 plate reader (Bio-Rad, Hercules, CA) according to the manufacturer’s instructions. Quantification of cytokines was performed by regression analysis from a standard curve generated from cytokine standards included in the kit with a lower limit of detection of 10 pg/ml.

### RNA Isolation

Age-matched four-month old male mice of PDE3B^−/−^and WT strains were CO_2_ euthanized and the epididymal white adipose tissue (WAT) was surgically removed. The WAT depots were stored in RNAlater solution (Ambion, Inc.) at 4 °C. When ready to use, the WAT was transferred into TriPure mRNA isolation reagent (Boeringer Manheim), followed by RNeasy Midi-kit isolation procedures (Qiagen, Inc.). A NanoDrop-1000 spectrophotometer test was then conducted to assess the RNA 260/280 purity and concentrations. The RNA was further tested for quality control using the Agilent 2100 BioAnalyzer. The purified RNA was later stored in −80 °C.

### PCR Array Gene Analysis

Total RNA (~500 ng) collected from the mice WAT depots were used in the 96-well RT^2^ Profiler PCR Array on mouse inflammasome related genes (SABiosciences). The PCR experiment was done in a 2-step process. The first step was to reverse-transcribe the total RNA into cDNA mixes using the RT^2^ First-Strand kit (SABiosciences). The RT^2^ SYBR Green/ROX PCR master mix was used with the cDNA and plated into the inflammasome array. The PCR reactions were conducted in the 7900HT standard block PCR machine (Applied Biosystems). After initial analysis with the predesigned array, we assembled a custom PCR array template, and purchased the custom array from SABiosciences. The experiments were replicated using the same PCR reagent kits as for the predesigned inflammasome array experiments.

### Real-time quantitative PCR (qPCR) assays

Total RNA was diluted to 10 ng/μl and 100 ng of RNA were subjected (in duplicate) to Real-time quantitative RT-PCR on the HT7900 Sequence Detection System (Applied Biosystems) by using QuantiTect SYBR Green RT-PCR kit (Qiagen) according to manufacturer’s protocols. The value of the target gene was normalized by that obtained from cyclophilin A, which served as the internal control. The ratio of the individual normalized value of KO (or WT) mice to the average of normalized values of WT (or KO) mice was calculated and the average was defined as an arbitrary unit. The sequences of primers are listed in Table S2.

### High-fat diet (HFD) studies

Age-matched (2-month old) WT and KO mice were housed two per cage with food and water *ad libitum*. The mice were fed high-fat diets (D12492 Research Diets, NJ) and low-fat standard diet (SD) (D12450B, Research Diets), respectively, for 10–14 weeks. Protein, carbohydrate, and fat contents as a percentage of caloric content were 20, 70, and 10 kcal% for low-fat, and 20, 20, and 60 kcal% for high-fat diets, respectively. Body weights were measured 1 times a week. The number of mice in each group was 6–7.

### Preparation of homogenates

Fresh mouse WAT from SvJ129 male WT and PDE3B^−/−^mice (8–16 weeks old) was collected and homogenized (1/3, w/v) in Buffer A [50 mM Hepes pH 7.4, 1 mM EDTA, 1 mM EGTA, 50 mM sucrose, 50 mM NaCl, 1 mM DTT, protease Inhibitor Cocktail and Phosphatase Inhibitor Cocktail (Thermoscientific, Rockford, IL)], using a Dounce glass homogenizer (20 strokes on ice), and centrifuged (500 xg, 15 min, 4 °C). To prepare total adipose tissue extracts (homogenates), pellets were resuspended in Buffer A, rehomogenized, and centrifuged (500 xg, 15 min, 4 °C). Supernatants (500 xg) were pooled, sonicated (on ice, 20 pulses, 40% duty cycle, output scale 4) in buffer A containing 1% (v/v) Triton-X100, and incubated with rotation (4 °C, 1 h) before centrifugation (15,000 xg, 20 min, 4 °C). These supernatants (designated as total adipose tissue extracts or homogenates) were used for protein measurements, PDE assays, or comparative protein expression analysis by Western immunoblotting (using samples of WT and PDE3B^−/−^WAT homogenates).

### Western Blotting

Equivalent amounts of total adipose tissue homogenates or lysates (usually 30 μg/lane) were subjected to SDS-PAGE using Tris-Glycine Gels (Invitrogen). Separated proteins were transferred to nitrocellulose membranes (Invitrogen). The membranes were incubated (4 °C, overnight) with blocking buffer in Dulbecco’s PBS (DPBS) containing 5% (w/v) non-fat dry milk (NFDM), and then with the appropriate primary antibody in blocking buffer (usually for 2-4 h, but sometimes overnight at 4 °C, depending on quality and sensitivity of the antibody). After incubation with primary antibody, membranes were washed in PBS (3 × 5 min), and incubated (2 h) with HRP (horseradish peroxidase)-labelled secondary antibody (Pierce) and washed with PBS (3 × 5 min). Immunoreactive proteins (membranes) were incubated with SuperSignal^®^ Westpico or Westfemto chemiluminescent reagents; signals were detected with an ImageQuant LAS4000 (GE Healthcare) image reader. Band densitometry was measured with Multi Gauge V2.3 software and the resultant individual values of target homogenate proteins were normalized by the values for HSP90 or GAPDH.

### Statistical analysis

Data are expressed as mean ± SE. All data presented with statistical analyses were analyzed using a Microsoft Excel 2010 Student’s *t* test with Data Analysis Toolpak (Microsoft). *P* values less than 0.05 were considered to be significant.

## Additional Information

**How to cite this article**: Ahmad, F. *et al.* Phosphodiesterase 3B (PDE3B) regulates NLRP3 inflammasome in adipose tissue. *Sci. Rep.* **6**, 28056; doi: 10.1038/srep28056 (2016).

## Supplementary Material

Supplementary Information

## Figures and Tables

**Table 1:** Levels of inflammatory cytokines (IL1β, TNF-α, MCP1, MIP-1β and IL-12) in WT and PDE3B^−/−^mice sera following challenge with LPS for 0 h, 6 h and 24 h.

Cytokines	WT			PDE3B^−/−^		
	0 h	LPS-6 h	LPS-24 h	0 h	LPS-6 h	LPS-24 h
IL1β	177+/−61	571+/−9	206+/−13	210+/−94	399+/−43	137+/−13
TNFα	14+/−4	615+/−34	72+/−8	9+/−1	325+/−44	89+/−12
MCP-1	19+/−6	29303+/−799	8470+/−754	20+/−16	10248+/−1289	3469+/−469
MIP-1β	45+/−4	6836+/−727	493+/−17	66+/−9	1653+/−289	272+/−27
IL12-p40	141+/−12	17429+/−929	441+/−112	244+/−96	7459+/−2402	444+/−253
IL6	39+/−30	6029+/−372	477+/−112	15+/−5	5667+/−2243	325+/−39
IL10	140+/−49	513+/−31	259+/−40	150+/−40	456+/−122	202+/−18
GMCSF	8+/−2	32+/−3	28+/−10	22+/−17	24+/−11	56+/−25

Serum cytokine concentrations (pg/mL) in wild-type (WT) and PDE3B^−/−^ mice treated with LPS for 0 h, 6 h and 24 h were compared with untreated controls. Serum samples from 4 mice per time point/group. Paired serum samples from untreated controls (Control), and mice treated with LPS for 6 h (LPS-6h) or 24 hr (LPS-24 h) previously stored at −80 °C, were analyzed using the Beadlyte Mouse Multi-Cytokine Detection System (Upstate Biotechnology, Lake Placid NY) and the Luminex100 plate reader (Luminex Corporation, Austin, TX) according to manufacturer’s instructions. Quantification of cytokines was performed by regression analysis from a standard curve generated from cytokine standards included in the kit with a lower limit of detection of 10 pg/ml for all cytokines evaluated. n = 2 independent experiments, each with samples from 4 WT and 4 PDE3B^−/−^ mice. Data presented as mean+/−SE.
