# Supplementary material for: Phosphodiesterase 3B (PDE3B) regulates NLRP3 inflammasome in adipose tissue
Source: Sci Rep. 2016 Jun 20;6:28056. doi: 10.1038/srep28056 (PMC4913246; doi:10.1038/srep28056)
Supplement: Supplementary Information [file srep28056-s1.doc]

**Phosphodiesterase 3B (PDE3B) regulates NLRP3 inflammasome in adipose tissue**

Faiyaz Ahmad^1,3^, Youn Wook Chung^1,3^, Yan Tang^1^, Steven C Hockman^1^, Shiwei Liu^1^, Yusuf Khan^1^, Kevin Huo^1^, Eric Billings^2^, Marcelo J Amar^1^, Alan T Remaley^1^, Vincent C. Manganiello^1^.

^1^Laboratory of Biochemical Physiology, Cardiovascular and Pulmonary Branch, ^2^The laboratory of Computational Biology, National Heart, Lung, and Blood Institute, National Institutes of Health, Bethesda, MD 20892, USA

^3^These authors contributed equally to this work.

**Running title:** PDE3B regulates NLRP3 inflammasome

**To whom correspondence may be addressed:** Faiyaz Ahmad, Cardiovascular and Pulmonary Branch, NHLBI/NIH, Building 10, Room 5N307, Bethesda, MD 20892-1434, USA Tel: (301) 496-3057, Fax: (301) 402-1610, E-mail: AhmadF@NHLBI.NIH.gov

Keywords: phosphodiesterases, PDE3B, inflammation, cAMP, PKA, NLRP3, TNFα, LDL receptor, NFκB, MAP kinase, apo-E.

**Figure S1.**

**Figure S1. NLRP3 inflammasome in adipose tissue.** Figure shows the role of PDE3B in mediating the activation of NLRP3 inflammasome. cAMP generated from activated adenylate cyclase activates protein kinase A (PKA). PDE3B reduces the concentration of cAMP, which results in decreased PKA activity. NLRP3 activators trigger inflammasome assembly by wide variety of agents (e.g. ROS, ATP, high glucose, bacteria, virus, PAMP/DAMP). The inactive procaspase-1 contains a CARD-containing prodomain. NLRP3 oligomerization and binding of procaspase-1 and ASC [apoptosis-associated speck-like protein containing a CARD] leads to formation of inflammasome. The proteolytic cleavage of procaspase-1 leads to activation of caspase-1. Active caspase-1 is capable of cleaving pro-1L-1β. These mature cytokines regulate a variety of cellular events by activating key processes such as the nuclear factor κB (NFkB) and mitogen-activated protein kinase (MAPK) pathways.  Increased adipocyte cAMP in PDE3B-/- mice decreases NLRP3 inflammasome **^1, 2^.** Increased expression of NOS2 through activation of AMP kinase in PDE3B-/- mice may also inhibit caspase-1 and prevent IL1β release**^3^**. LRR: leucine-rich repeat; NACHT: nucleotide-binding, and oligomerization domain; PYD: pyrin domain; ROS: Reactive oxygen species; PAMP: Pathogen Assocated Molecular Patterns; DAMP: Damage Associated Molecular Patterns.

**Methods**

**Microarray analysis.** RNA isolation from WAT of 4 WT and 4 PDE3B-/- mice (7-months) was performed as described previously ^4^. cRNA was synthesized with BioArray High Yield RNA Transcript Labeling Kit (T7) (Enzo Life Sciences, Inc., Farmingdale, NY), purified via GeneChip Sample Cleanup Module (Affymetrix, Santa Clara, CA), and fragmented cRNA (15 µg) was prepared for hybridization cocktails with GeneChip Eukaryotic Hybridization Control Kit (Affymetrix). Samples were applied to Affymetrix MU74A Version 2 chips, hybridized (16 h), stained, washed and scanned. Gene expression values were estimated using both Affymetrix Microarray Suite (MAS 5.1) and Robust Multi Array average (RMA) software. Of the 12488 transcripts on the array, 7298 genes were identified as ‘Present’ by MAS 5.1 in any one of the samples. Mean expression values for this subset of genes were then combined for the four WT and PDE3B-/- biological replicates. A total of 573 genes were identified as significant at the *p*≤0.05 level using a Welch’s *t*-test with a Benjamin-Hochberg false-discovery rate for multiple testing correction (Table S1). Of these genes, 195 had greater than a two-fold increase (35 genes) or decrease (160 genes) between PDE3B-/- and WT mice. The focused set was clustered and organized by principal components analysis using GeneSpring 7.3. The focused list of 195 genes as well as a less stringent list of 401 genes that met selection criteria as ‘Present’, and were statistically significant with a false discovery rate <0.05 but a minimum fold-change of 1.5, were analyzed with MetaCore from GeneGO Inc., version 4.3 build 9311 and Ingenuity Pathway. These analyses identified significant changes in expression of genes involved in the pathways regulating the Tricarboxylic Acid (TCA) cycle, CCR3 chemokine receptor-signaling, and antigen presentation, as well as fatty acid oxidation (FAO) and mitochondrial oxidative phosphorylation. The TCA cycle pathway was significantly associated (*p*≤10^-16.5^) with changes in gene expression in PDE3B-/- WAT. Of 20 transcripts which may be associated with the TCA cycle, expression of 11 transcripts was increased in PDE3B-/- WAT.

**Crossbreeding of C57BL/6 PDE3B-/- mice.** PDE3B knockout mice in C57BL/6 background were described in Guirgus, E. et al. ^5^. ApoE-/-/PDE3B-/- and LDL-R-/-/PDE3B-/- were crossbred in C57BL/6 background. PDE3B-/- mice were mated with ApoE knockout mice from Jackson Laboratory (strain name: B6.129P2-ApoE tm1 Unc/J, stock # 002052). The resulting heterozygous offspring for both the PDE3B and ApoE genes were verified by PCR. The ApoE protocol for PCR is <https://www2.jax.org/protocolsdb/f?p=116:5:0::NO:5:P5_MASTER_PROTOCOL_ID,P5_JRS_CODE:221,002052>

The PDE3B PCR protocol uses a combination of two sets of primers.

1. Primer set to verify the PDE3B knockout allele

Forward 5’-ACACACCCCACAAAACACACCGTTGCACAGAACA-3’

Reverse 5’-AAAGCGCCTCCCCTACCCGGTAGAATTGACCTGCA-3’

are used to produce an amplified fragment of 750 base pairs.

1. Primer set to verify the Wild type allele

Forward 5’-ACACACCCCACAAAACACACCGTTGCACAGAACA-3’

Reverse 5’-CACGTTGCAGAAGCGGCAGAGGTGGAAGAAG– 3’

are used produce an amplified fragment of 1500 base pairs.

The conditions for PCR are to denature at 94°C for 5min. then 30 cycles of 94°C for 45 sec, 63°C for 45 sec, 68°C for 4min. This is finally followed with a final extension at 68°C for 10min. The reaction mixture is composed according to Clontech Advantage GC cDNA PCR kit using the provided instructions (Catalog # 639115). One ul of genomic DNA is used as the template DNA for the PCR reaction. Subsequent mating of heterozygous offspring finally yielded mice with both of the genes deleted. Mating from double knockout offspring yielded a final line of all offspring with both genes deleted (PDE3B-/-/ApoE-/-).

In order to obtain mice with both PDE3B and LDL-R genes knocked out, PDE3B-/- mice in C57BL/6 background as previously mentioned^5^ were used to mate with LDL-R-/- mice from Jackson Laboratory (Strain: B6.129S7-Ldlr tm1 Her/J, stock #002207). The resulting heterozygous offspring for both the PDE3B and the LDL-R genes were verified by PCR. The PDE3B PCR protocol was the same as mentioned above for the PDE3B-/-/ApoE-/- mice. Subsequent mating of heterozygous offspring finally yielded mice with both of the genes deleted. Mating from double knockout offspring yielded a final line of all offspring with both genes deleted (PDE3B-/-/LDL-R-/-).

The LDL-R Knockout specific PCR protocol uses a combination of two sets of primers.

1. Primer set to verify the knockout allele

Forward 5'-CCATATGCATCCCCAGTCTT-3'

Reverse 5'-AATCCATCTTGTTCAATGGCCGATC-3'

are used to produce a 350 base pair fragment.

1. Primer set to verify the wild type allele

Forward 5'-CCATATGCATCCCCAGTCTT-3'

Reverse 5'-GCGATGGATACACTCACTGCTAC-3'

are used to produce a 167 base pair fragment.

The conditions for PCR are to denature at 94°C for 2min. then 35 cycles of a three stage amplification at 94°C for 30 sec, 65°C for 30 sec, and 72°C for 45 sec, followed with a final extension at 72°C for 7 min. The reaction mixture is composed with the use of 1 unit of Platinum Taq Polymerase (Invitrogen, Cat.# 10966-018 ), 1X final concentration of Platinum Taq Reaction buffer, 200nM final concentration of primers, 2mM of MgCl2 final concentration, 200 uM dNTP mixture final concentration in a 30 ul reaction volume including 5 ul of genomic DNA as template.

**Supplementary Table S1. Affymetrix Microarray**

Table S1. Continued

Table S1. continued

Table S1. continued

A total of 573 genes were identified as significant at the p≤0.05 level using a Welch’s t-test with a Benjamini-Hochberg false discovery rate for a multiple testing correction. Of these genes, one hundred ninty five had greater than a two-fold increase (35 genes) or decrease (160 genes) between the KO and WT mice.

**Table S2.**

primers

| **Official Symbol** | **Accession No** | **Left primer (5'-3')** | **Right primer (5'-3')** |
| --- | --- | --- | --- |
| Acadl | [BC027412](http://www.ncbi.nlm.nih.gov/entrez/viewer.fcgi?db=nucleotide&val=20071666) | gcttcagcctccactcagat | ggctatggcaccgatacact |
| Acadvl | [NM_017366](http://www.ncbi.nlm.nih.gov/entrez/viewer.fcgi?db=nucleotide&val=23956083) | tctgtccagagcctcaaggt | agcctcaatgcaccagctat |
| Adam8 | [NM_007403](http://www.ncbi.nlm.nih.gov/entrez/viewer.fcgi?db=nucleotide&val=6680641) | gacctctctccaaggcacag | atgacggcaacttcttgtcc |
| Adrb1 | [NM_007419](http://www.ncbi.nlm.nih.gov/entrez/viewer.fcgi?db=nucleotide&val=6680665) | atcgttctgctcatcgtggt | atgaagaggttggtgagcgt |
| Adrb3 | [NM_013462](http://www.ncbi.nlm.nih.gov/entrez/viewer.fcgi?db=nucleotide&val=7304870) | acaggaatgccactccaatc | aaggagacggaggaggagag |
| Ccl2 | [NM_011333](http://www.ncbi.nlm.nih.gov/entrez/viewer.fcgi?db=nucleotide&val=6755429) | aggtccctgtcatgcttctg | cgttaactgcatctggctga |
| Ccl3 | [X12531](http://www.ncbi.nlm.nih.gov/entrez/viewer.fcgi?db=nucleotide&val=53122) | cctctgtcacctgctcaaca | gtagactcacatggcgctga |
| Ccr2 | [NM_009915](http://www.ncbi.nlm.nih.gov/entrez/viewer.fcgi?db=nucleotide&val=6753465) | ctcagttcatccacggcata | caaggctcaccatcatcgta |
| Ccr5 | [X94151](http://www.ncbi.nlm.nih.gov/entrez/viewer.fcgi?db=nucleotide&val=1419628) | gactagaccaggccatgcag | ccggaacttctctccaacaa |
| Cd68 | [NM_009853](http://www.ncbi.nlm.nih.gov/entrez/viewer.fcgi?db=nucleotide&val=6753351) | gcagcacagtggacattcat | tcaaggtgaacagctggaga |
| Cidea | [NM_007702](http://www.ncbi.nlm.nih.gov/entrez/viewer.fcgi?db=nucleotide&val=6680943) | ctcggctgtctcaatgtcaa | tccttaacacggccttgaac |
| Cox4i1 | [NM_009941](http://www.ncbi.nlm.nih.gov/entrez/viewer.fcgi?db=nucleotide&val=6753497) | agaaggcgctgaaggagaa | ctggatgcggtacaactgaa |
| Cpt2 | [NM_009949](http://www.ncbi.nlm.nih.gov/entrez/viewer.fcgi?db=nucleotide&val=6753513) | gctctaaggtatctggcagc | ctggtggacaggatgttgtg |
| Cyba | [NM_007806](http://www.ncbi.nlm.nih.gov/entrez/viewer.fcgi?db=nucleotide&val=22094076) | gtggactcccattgagccta | ctcctcttcaccctcactcg |
| Cybb | [NM_007807](http://www.ncbi.nlm.nih.gov/entrez/viewer.fcgi?db=nucleotide&val=31542439) | ggcacacattcacactgacc | gcttatcacagccacaagca |
| Dio2 | [AF096875](http://www.ncbi.nlm.nih.gov/entrez/viewer.fcgi?db=nucleotide&val=4206102) | tctgctcagtctgtggttgg | aggactccttgcaccatgac |
| Emr1 | [DQ167573](http://www.ncbi.nlm.nih.gov/entrez/viewer.fcgi?db=nucleotide&val=74275390) | agcatccgagacacacacag | atggccaaggcaagacatac |
| Icam1 | [NM_010493](http://www.ncbi.nlm.nih.gov/entrez/viewer.fcgi?db=nucleotide&val=30172560) | tggtgatgctcaggtatcca | ggtccactctcgagctcatc |
| Ncf2 | [NM_010877](http://www.ncbi.nlm.nih.gov/entrez/viewer.fcgi?db=nucleotide&val=31560561) | ctggctgaggccatcagact | aggccactgcagagtgcttg |
| Nrbf1 | [NM_025297](http://www.ncbi.nlm.nih.gov/entrez/viewer.fcgi?db=nucleotide&val=13384651) | tgctgtgaaaggatctgacg | gccatagttcccttggatca |
| Pde3b | AF547435.1 | ccaattcctggcttacctca | gcaatctgtccagaaccaag |
| Ppara | [NM_011144](http://www.ncbi.nlm.nih.gov/entrez/viewer.fcgi?db=nucleotide&val=53764) | agaccttgtgtatggccgag | actggcagcagtggaagaat |
| Ppargc1a | [NM_008904](http://www.ncbi.nlm.nih.gov/entrez/viewer.fcgi?db=nucleotide&val=6679432) | ccgagaattcatggagcaat | gtgtgaggagggtcatcgtt |
| Ppia | [NM_008907](http://www.ncbi.nlm.nih.gov/entrez/viewer.fcgi?db=nucleotide&val=6679438) | aaggtgaaagaaggcatgagc | agttgtccacagtcggaaatg |
| Rb1 | [NM_009029](http://www.ncbi.nlm.nih.gov/entrez/viewer.fcgi?db=nucleotide&val=6677678) | gcctcagccttccatactca | gaaggcgtgcacagagtgta |
| Slc25a20 | [BC052871](http://www.ncbi.nlm.nih.gov/entrez/viewer.fcgi?db=nucleotide&val=31127296) | ggacgtgctcaagtctcgat | tcggatcagctctctcaaca |
| Sod1 | [BC086886](http://www.ncbi.nlm.nih.gov/entrez/viewer.fcgi?db=nucleotide&val=56270594) | cagcatgggttccacgtcca | cacattggccacaccgtcct |
| Tnfa | [NM_013693](http://www.ncbi.nlm.nih.gov/entrez/viewer.fcgi?db=nucleotide&val=7305584) | atgcaccaccatcaaggact | gaggcaacctgaccactctc |
| Ucp1 | [NM_009463](http://www.ncbi.nlm.nih.gov/entrez/viewer.fcgi?db=nucleotide&val=31981931) | aactgtacagcggtctgcct | taagccggctgagatcttgt |
| Vcam1 | [NM_011693](http://www.ncbi.nlm.nih.gov/entrez/viewer.fcgi?db=nucleotide&val=31981429) | gcgagtcaccattgttctca | ctccatggtcagaacggact |

References:

1. Yan, Y. et al. Dopamine controls systemic inflammation through inhibition of NLRP3 inflammasome. *Cell* **160**, 62-73 (2015).

2. Lee, G.S. et al. The calcium-sensing receptor regulates the NLRP3 inflammasome through Ca2+ and cAMP. *Nature* **492**, 123-127 (2012).

3. Kim, Y.M., Talanian, R.V., Li, J. & Billiar, T.R. Nitric oxide prevents IL-1beta and IFN-gamma-inducing factor (IL-18) release from macrophages by inhibiting caspase-1 (IL-1beta-converting enzyme). *J Immunol* **161**, 4122-4128 (1998).

4. Choi, Y.H. et al. Alterations in regulation of energy homeostasis in cyclic nucleotide phosphodiesterase 3B-null mice. *J. Clin. Invest* **116**, 3240-3251 (2006).

5. Guirguis, E. et al. A role for phosphodiesterase 3B in acquisition of brown fat characteristics by white adipose tissue in male mice. *Endocrinology* **154**, 3152-3167 (2013).
